# The Fresh Air Mission

**Published:** 1890-07

**Authors:** 


### THE FRESH AIR MISSION.

THIS noble charity, under the direction of a few of Buffalo's public-spirited citizens, is doing a grand work in the way of providing that most potent medicine — fresh air — for those who are in the greatest need thereof and the least able to provide themselves with it. During the Summer of 1889, 360 children, twelve mothers, and one grandmother were sent to the country for a visit of two weeks each, and when needed by sickness some cases were kept out a much longer time. This season it is proposed to enlarge very much the usefulness of the mission in the direction named, and to this end it makes an appeal to every philanthropic man and woman in Buffalo for aid. There should be no lack of funds for such a worthy object, and this great city of wealth and prosperity should take an abiding interest in the success of the enterprise. To Miss Alice Moore and her associates and coadjutors in the noble work already accomplished all praise is due, and they must be sustained in their present efforts to carry on their hallowed mission towards greater usefulness.

Miss Alice Putnam Baker is a solicitor for the mission, and we understand she is meeting with considerable success in obtaining subscriptions to the fund. Contributions can also be sent to Mrs. Herman Mynter, treasurer, 195 Franklin street, Buffalo, who will duly acknowledge any monies received.

Subscribers who desire to become members of the association should be careful to state that fact. The following is the form of application for membership which has been distributed :

TO MRS. HERMAN MYNTER,

*Chairman Committee on Subscriptions,*

*195 Franklin street, Buffalo, N. Y. :*

I hereby apply for membership in the Fresh Air Mission of Buffalo for one year from the date of this application, and enclose one dollar, the annual fee.

. . . . . . . . . . . . . . . (Name)

. . . . . . . . . . . . . . (Address)

. . . . . . . (Date) . . . . . . . . . . . . . . . .

